# Generation of Nasal Cell‐Derived Human Alveolar Organoids and Organoid‐Macrophage Assembloids for in Vitro Lung Modeling

**DOI:** 10.1002/advs.77093

**Published:** 2026-08-11

**Authors:** Man Chun Chiu, Shuxin Zhang, Cun Li, Yifei Yu, Zhixin Wan, Jingjing Huang, Wei Xue, Ying Zhou, Xiaoxin Zhu, Qiaoshuai Lan, Jiali Wu, Zijun Zhao, Yuhong Liu, Xinjie Meng, Lin Huang, Hin Chu, Honglin Chen, Kelvin Kai‐Wang To, Kwok Yung Yuen, Jie Zhou

**Affiliations:** ^1^ Department of Microbiology School of Clinical Medicine Li Ka Shing Faculty of Medicine The University of Hong Kong Hong Kong China; ^2^ State Key Laboratory of Emerging Infectious Diseases The University of Hong Kong Hong Kong China; ^3^ Centre for Virology Vaccinology and Therapeutics Hong Kong Science and Technology Park Hong Kong China; ^4^ Pandemic Research Alliance Unit The University of Hong Kong Hong Kong China; ^5^ BiomOrgan Limited Hong Kong China

**Keywords:** alveolar macrophages, alveolar organoids, assembloids, influenza A viruses, nasal organoids

## Abstract

We established robust protocols to generate physiological and functional alveolar organoids (nsoAlvO) from readily accessible and expandable nasal cell‐derived organoids, and alveolar macrophages (monoAM) from peripheral blood monocytes. Through co‐culture of nsoAlvO and monoAM, we generated organoid‐macrophage assembloids, in which both components exhibited enhanced maturation. Comprehensive analyses, including immunostaining, functional assays, and single‐cell RNA sequencing, demonstrated that the nsoAlvO and monoAM phenotypically and functionally resemble their native counterparts and engage in dynamic and extensive epithelial‐macrophage communications. SSEA‐1+ club cells were identified as the primary alveolar progenitor cells for nsoAlvO. Influenza virus infection in nsoAlvO revealed differential replicative fitness of H5N1 and H1N1 viruses, which recapitulate their authentic tropism in vivo. Notably, the addition of monoAM reduced H5N1 and H1N1 infection in nsoAlvO, suggesting a protective effect of alveolar macrophages against virus dissemination. These human alveolar organoids and organoid‐macrophage assembloids provide universally accessible and physiologically relevant in vitro lung models for biomedical research and translational medicine.

## Graphical abstract

This study demonstrates the generation of human alveolar organoids from nasal cells, which, when co‐cultured with monocyte‐derived macrophages, promotes maturation of both components and forms alveolar assembloids resembling native alveoli. These alveolar organoids and assembloids offer accessible, physiologically relevant in vitro systems for advancing lung research and translational applications.

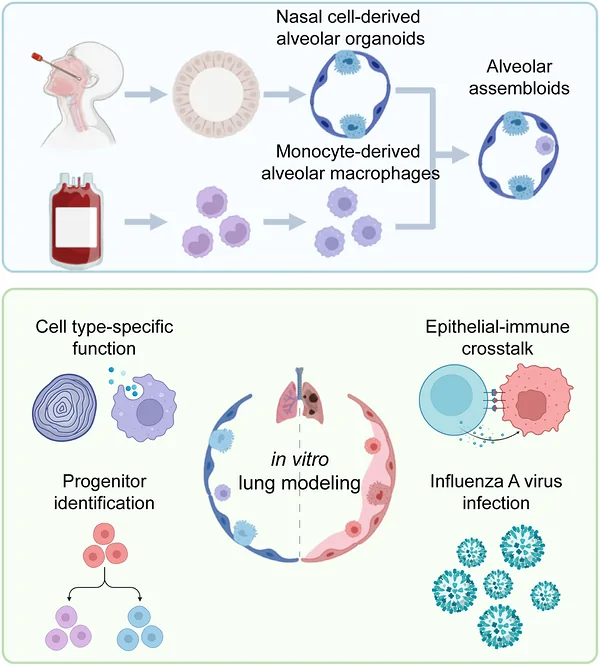

## 1 Introduction

The human respiratory tract, which is lined with airway and alveolar epithelium, is the primary site of infection. The alveolar epithelium comprises alveolar type 1 (AT1) and type 2 (AT2) epithelial cells. While thin and flat AT1 cells provide an ideal interface for gas exchange, cuboidal AT2 cells synthesize, secrete, and recycle pulmonary surfactants to regulate surface tension and alveolar stability. AT2 cells are also known as facultative stem cells, which proliferate and differentiate into AT1 cells [1]. In addition to epithelial cells, alveolar macrophages (AM) reside within the alveoli and play an essential role in innate immunity against invading pathogens and external stimuli [2, 3].

A physiologically relevant and readily accessible in vitro model of human alveoli is crucial for studying lower respiratory infections. Alveolar and/or distal lung organoids have been established from adult stem cells (ASC) [4, 5, 6, 7, 8] or pluripotent stem cells (PSC) [9, 10, 11, 12, 13, 14, 15, 16, 17] in recent years; some PSC‐derived organoids contained isogenic macrophages [13, 14, 15, 16, 17]. PSC‐derived organoids are favorable models for developmental biology research, yet their intrinsic fetal‐like properties may limit their utility for modeling post‐birth diseases such as respiratory infections. ASC‐derived organoids are phenotypically more mature than their PSC counterparts, but existing ASC‐derived alveolar organoids lack immune cell components.

We previously established an ASC‐based, two‐phase, bi‐potential human respiratory organoid culture system, which enables the generation of long‐term expandable organoids from lung tissues and differentiation into mature organoids with high efficiency. The organoids could consecutively propagate for 6–12 months. With well‐defined protocols, the organoids undergo proximal differentiation into airway organoids (AwO) that comprise ciliated, goblet, basal, and club cells [18, 19, 20, 21]; or distal differentiation into alveolar organoids (AlvO) that comprise AT1 and AT2 cells [7, 8]. Yet, most research laboratories may not have access to fresh primary lung tissues. To overcome limited tissue availability, we generated long‐term expandable nasal cell‐derived organoids (NsO) using nasal epithelial cells obtained non‐invasively from healthy volunteers via nasal swabs. Upon induction of maturation, differentiated nasal organoids accommodate four major airway epithelial cell types similar to airway organoids, yet they retain the attributes of the upper airway epithelium [22].

We noted that club cells, which possess intrinsic alveolar differentiation capacity and contribute to alveolar regeneration in vivo [23, 24, 25, 26, 27, 28], are maintained in the NsO during long‐term expansion. Based on these findings, we hypothesized that nasal cell‐derived organoids may potentially differentiate into alveolar organoids (i.e., nasal‐derived alveolar organoids, nsoAlvO). We further hypothesized that co‐culturing alveolar organoids with peripheral monocytes may generate biologically relevant alveolar macrophages (i.e., monocyte‐derived alveolar macrophages, monoAM). Overall, we developed novel alveolar assembloids comprising nsoAlvO and monoAM for modeling the native human alveoli and investigating viral infections.

## 2 Results

### 2.1 Derivation and Characterization of Nasal‐Derived Alveolar Organoids

To develop nasal‐derived alveolar organoids, we induced alveolar differentiation by incubating the undifferentiated nasal cell‐derived organoids (NsO) in a distal differentiation (DD) medium composed of WNT agonist (CHIR99021) and other alveolar maturation additives (including dexamethasone, 8‐bromo‐cAMP, IBMX) (Figure 1A) [7, 8, 23]. Dextran sulfate (ds), which activates the WNT signaling pathway and inhibits adhesion molecules [29], was added to the DD medium (i.e., DD+ds medium) in the first week to prevent cellular aggregation during suspension culture. The organoids differentiated sequentially in the DD+ds medium in the first week, followed by the DD medium in the second week of differentiation culture (Figure 1A), eventually formed cystic organoids with thin walls and large lumens resembling the morphology of alveoli, i.e., nasal‐derived alveolar organoids (nsoAlvO) (Figure 1B). We then confirmed the presence of thin and flat AT1‐like cells (Figure 1C) and cuboidal AT2‐like cells with characteristic lamellar bodies (Figure 1D) in nsoAlvO by transmission electron microscopy.

**FIGURE 1:**
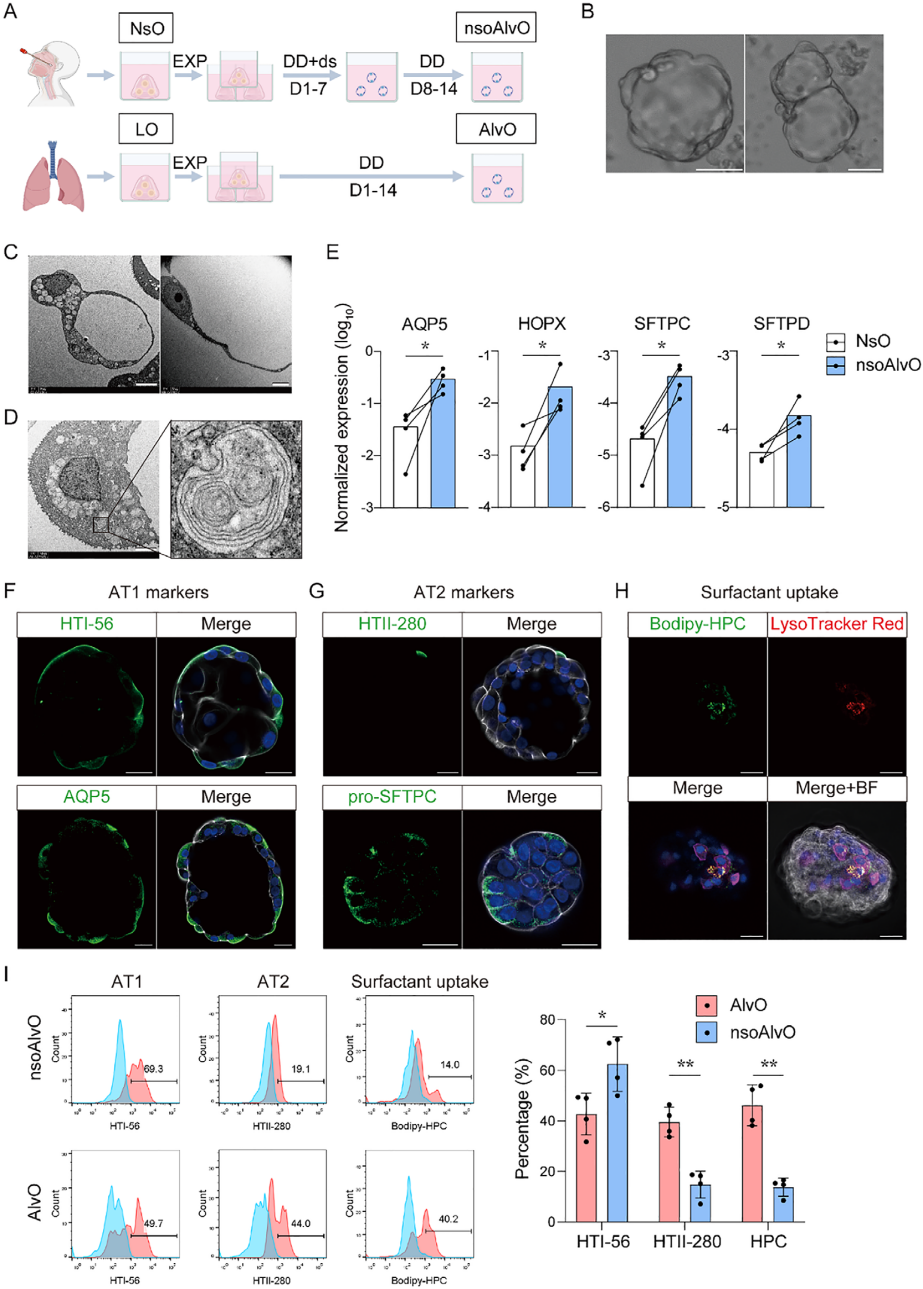
Derivation and characterization of nasal‐derived alveolar organoids. (A) Schematic overview of the expansion of nasal cell‐derived (NsO) or lung tissue‐derived organoids (LO) in matrigel using expansion (EXP) medium and subsequent differentiation in suspension using distal differentiation (DD) medium with or without dextran sulfate (ds) to generate nasal‐derived (nsoAlvO) or tissue‐derived alveolar organoids (AlvO). (B) Bright‐field images of nsoAlvO. Scale bar: 50 µm. (C,D) Ultrastructural images of (C) AT1‐like cells or (D) AT2‐like cells with lamellar bodies in nsoAlvO. Scale bar: 5 µm for (C); 2 µm for (D). (E) Expression of AT1 and AT2 marker genes in parental NsO and differentiated nsoAlvO. N = 4 independent biological replicates. Ratio paired t‐tests. (F‐G) Confocal images of nsoAlvO labeled with (F) AT1 markers HTI‐56 and AQP5 (green) or (G) AT2 markers HTII‐280 and pro‐SFTPC (green), counterstained with DAPI (blue) and Phalloidin (white). Scale bar: 20 µm. (H) Live cell confocal images of nsoAlvO incubated with Bodipy‐HPC (green) and LysoTracker Red (red), counterstained with Hoechst (blue) and CellMask plasma membrane stain (violet), overlaid with the bright‐field images. Scale bar: 20 µm. (I) Flow cytometry analysis of the abundance of HTI‐56+ AT1‐like and HTII‐280/Bodipy‐HPC+ AT2‐like cells in nsoAlvO and AlvO. Representative histograms are shown on the left. Data on the right represent mean ± SD, N = 4 independent biological replicates. Unpaired t‐tests.

In addition to morphological resemblance to alveoli, the nsoAlvO showed significantly upregulated expression levels of AT1 marker genes (AQP5, HOPX) compared to the parental undifferentiated organoids (Figure 1E). SFTPC and SFTPD, the definitive AT2 markers [30], were also significantly upregulated in nsoAlvO compared to the parental undifferentiated organoids (Figure 1E). The expression of these AT1 and AT2 markers in nsoAlvO reached a level comparable to that in lung tissue‐derived alveolar organoids (AlvO) (Figure S1A). We identified AT1‐ and AT2‐like cells in nsoAlvO by immunostaining (Figure 1F,G) using well‐established AT1‐specific markers HTI‐56/AQP5 and AT2‐specific markers HTII‐280/pro‐SFTPC [6, 30, 31, 32]. The nsoAlvO exhibited an apical‐out polarity as shown by the exterior distribution of apical‐localizing proteins, including AQP5, HTI‐56, and HTII‐280 (Figure 1F,G), due to suspension culture [7, 20, 33, 34]. Importantly, AT2‐like cells in nsoAlvO were capable of uptaking and storing fluorescent pulmonary surfactant components in lysotracker red‐labeled lamellar bodies (Figure 1H), which functionally resembled the native AT2 cells. The nsoAlvO consisted of approximately 50%–70% HTI‐56+ AT1‐like and 5%–20% HTII‐280/Bodipy‐HPC+ AT2‐like cells as determined by flow cytometry analysis (Figure 1I and Figure S1B). Yet there were significantly more AT1‐like and fewer AT2‐like cells in nsoAlvO than AlvO (Figure 1I). The significant upregulation of cell type‐specific markers and presence of abundant AT1‐, AT2‐like cells in nsoAlvO upon differentiation culture were validated across four independent biological replicates (Figure 1E,I). Thus, we developed a robust protocol to generate alveolar organoids from nasal cell‐derived organoids. The resultant alveolar organoids morphologically and functionally mimic the native alveolar epithelium. The major advancement over previously established alveolar organoids is the utilization of easily obtainable nasal epithelial cells as cell sources.

### 2.2 Derivation and Characterization of Monocyte‐Derived Alveolar Macrophages

Alveolar macrophages (AM) are the most abundant innate immune cells residing within the alveoli [2, 3]. They are a self‐sustaining, tissue‐residing population, although the blood‐circulating monocytes repopulate the alveolar macrophage niche when the original population is depleted or damaged [3, 35, 36, 37]. Alveolar macrophages are normally isolated from bronchoalveolar lavage fluid or lung tissues, which are not readily accessible for research due to invasive acquisition procedures, tissue availability, and limited proliferative capacity in vitro [38]. An in vitro model of human AM remains elusive. Given that the crosstalk between alveolar epithelial cells and macrophages was essential for the self‐renewal and differentiation of both components [39], we inferred that incubating monocyte‐derived macrophages (MDM) in the DD medium supplemented with AM induction molecules, or co‐culturing MDM with nsoAlvO, might provide an alveolar‐like niche for generating biologically relevant AM‐like cells.

We isolated monocytes from human peripheral blood and generated MDM (Figure 2A) as described previously [40]. Based on previous studies, GM‐CSF and TGFβ promote the differentiation and homeostasis of alveolar macrophages [39, 41, 42, 43, 44, 45, 46, 47, 48], while WNT signaling induces inflammation and restricts their proliferation [49]. Thus, we incubated MDM in a modified DD medium without WNT agonist CHIR99021 and supplemented with GM‐CSF, TGFβ, DAPT, and Y‐27632 (mDD medium afterward) for two weeks, to generate monocyte‐derived alveolar macrophages (monoAM) (Figure 2A). The monoAMs showed elevated expression levels of human AM marker genes PPARγ, APOC1, and APOE [36, 50, 51] compared to the parental monocyte or MDM (Figure 2B). Immunostaining revealed that most monoAMs expressed CD169 and CD206 (Figure 2C), which are specific markers of human alveolar macrophages [36, 52, 53, 54, 55]. Flow cytometry analysis indicated approximately 60–80% of monoAMs were autofluorescent (Figure 2D), an attribute of alveolar macrophages as documented previously [56, 57, 58]. Consistently, monoAMs exhibited significantly more abundant CD169+ or CD206+ cells compared to the parental monocyte and MDM (Figure 2D). The significant upregulation of AM‐specific marker genes and proteins in monoAM was validated across four independent biological replicates (Figure 2B,D).

**FIGURE 2:**
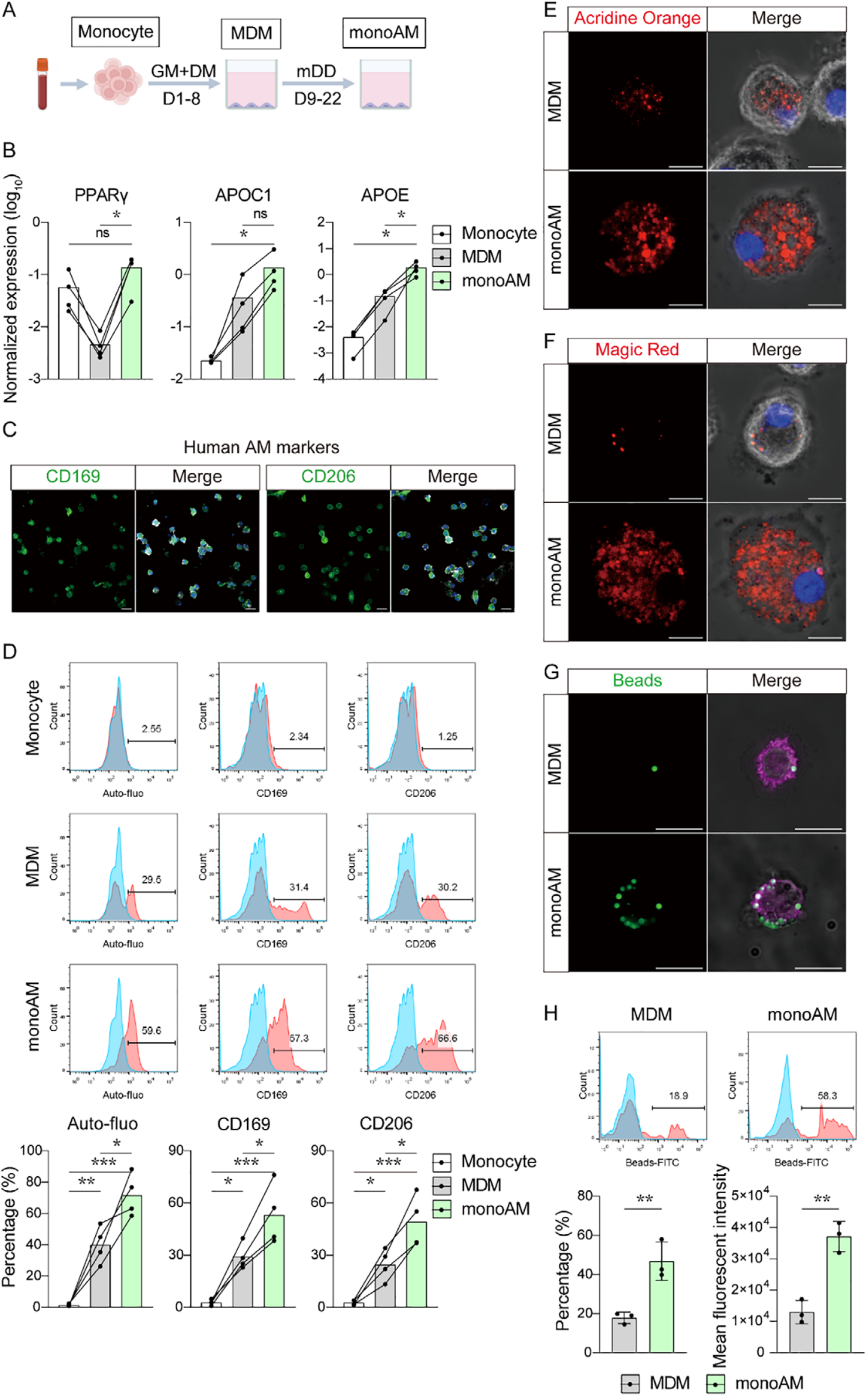
Derivation and characterization of monocyte‐derived alveolar macrophages. (A) Schematic overview of the sequential differentiation of monocyte into monocyte‐derived macrophages (MDM) using growth (GM) and differentiation medium (DM). Subsequently, the differentiation of MDM into monocyte‐derived alveolar macrophages (monoAM) using mDD medium. (B) Expression of alveolar macrophage marker genes. N = 4 independent biological replicates. RM one‐way ANOVA with multiple comparisons. (C) Confocal images of monoAM labeled with AM markers CD169 or CD206 (green), counterstained with DAPI (blue) and Phalloidin (white). Scale bar: 20 µm. (D) Flow cytometry analysis of the abundance of auto‐fluorescent or CD169/CD206+ AM‐like cells. Representative histograms are shown on top. N = 4 independent biological replicates. RM one‐way ANOVA with multiple comparisons. (E–G) Live cell confocal images of macrophages incubated with (E) acridine orange (red), (F) magic red (red), or (G) fluorescent microbeads (green), counterstained with Hoechst (blue) and overlaid with the bright‐field images. Scale bar: 10 µm. (H) Flow cytometry analysis of macrophages incubated with fluorescent microbeads. Representative histograms are shown on top. Data represent mean ± SD, N = 3 independent biological replicates. Unpaired t‐tests.

Alveolar macrophages normally maintain high phagocytic activity, produce low levels of inflammatory cytokines, and are generally immunosuppressive [48, 59]. We showed more abundant lysosomes in monoAM than MDM by live cell imaging of acridine orange‐labeled acidic vesicles (Figure 2E). Magic red is a substrate that produces fluorescent signals upon cleavage by cathepsin B. Live cell imaging of magic red‐treated monoAMs showed higher proteolytic activity compared to MDM (Figure 2F). The monoAMs invariably showed higher phagocytic activity than MDM, as indicated by uptake of fluorescent latex beads in live cell imaging and flow cytometry (Figure 2G,H). Interestingly, monoAMs expressed higher levels of interferons (IFNα, IFNβ, IFNλ) and lower levels of inflammatory cytokines and chemokines (IL6, IP10, RANTES) than MDM (Figure S2). Thus, we developed a robust protocol to direct the differentiation of peripheral blood circulating monocytes toward functional alveolar macrophages. The results indicated that the mDD medium alone, supplemented with factors for alveolar macrophage self‐renewal and differentiation, was sufficient to induce MDM differentiation into AM‐like cells.

### 2.3 Generation and In‐Depth Characterization of Alveolar Organoid‐Macrophage Assembloids

We used DD‐based media, i.e., mDD medium for monoAM, and DD medium for nsoAlvO, aiming for the ultimate co‐culture of organoids and macrophages in the same medium to establish an alveolar organoid‐macrophage assembloid model. First, we used transwell inserts to co‐culture nsoAlvO and monoAM in the upper and bottom chambers, respectively, with the mDD medium for 5–7 days (Figure 3A), which enabled easy evaluation of each component subsequently. We examined the nsoAlvO and monoAM with or without co‐culture. These indirectly co‐cultured nsoAlvO appeared stable and viable, with abundant HTI‐56+ AT1‐like and pro‐SFTPC+ AT2‐like cells (Figure 3B). The co‐cultured nsoAlvO contained significantly more abundant HTI‐56+ AT1‐like and HTII‐280+ AT2‐like cells than the nsoAlvO alone in DD or mDD medium (Figure 3C). Meanwhile, the co‐cultured monoAMs showed more abundant autofluorescent and CD169/CD206+ cells compared to monoAM alone (Figure 3D). In addition to indirect co‐culture using transwell inserts, we performed direct co‐culture by incubating organoid suspension with macrophages in the same culture compartment (Figure 3A). Immunostaining confirmed the incorporation of CD206+ monoAM into nsoAlvO to form assembloids (Figure 3E). Collectively, the nsoAlvO and monoAM adapt to co‐culture, establish cell‐cell contact, and provide a favorable niche mutually for improved maturity.

**FIGURE 3:**
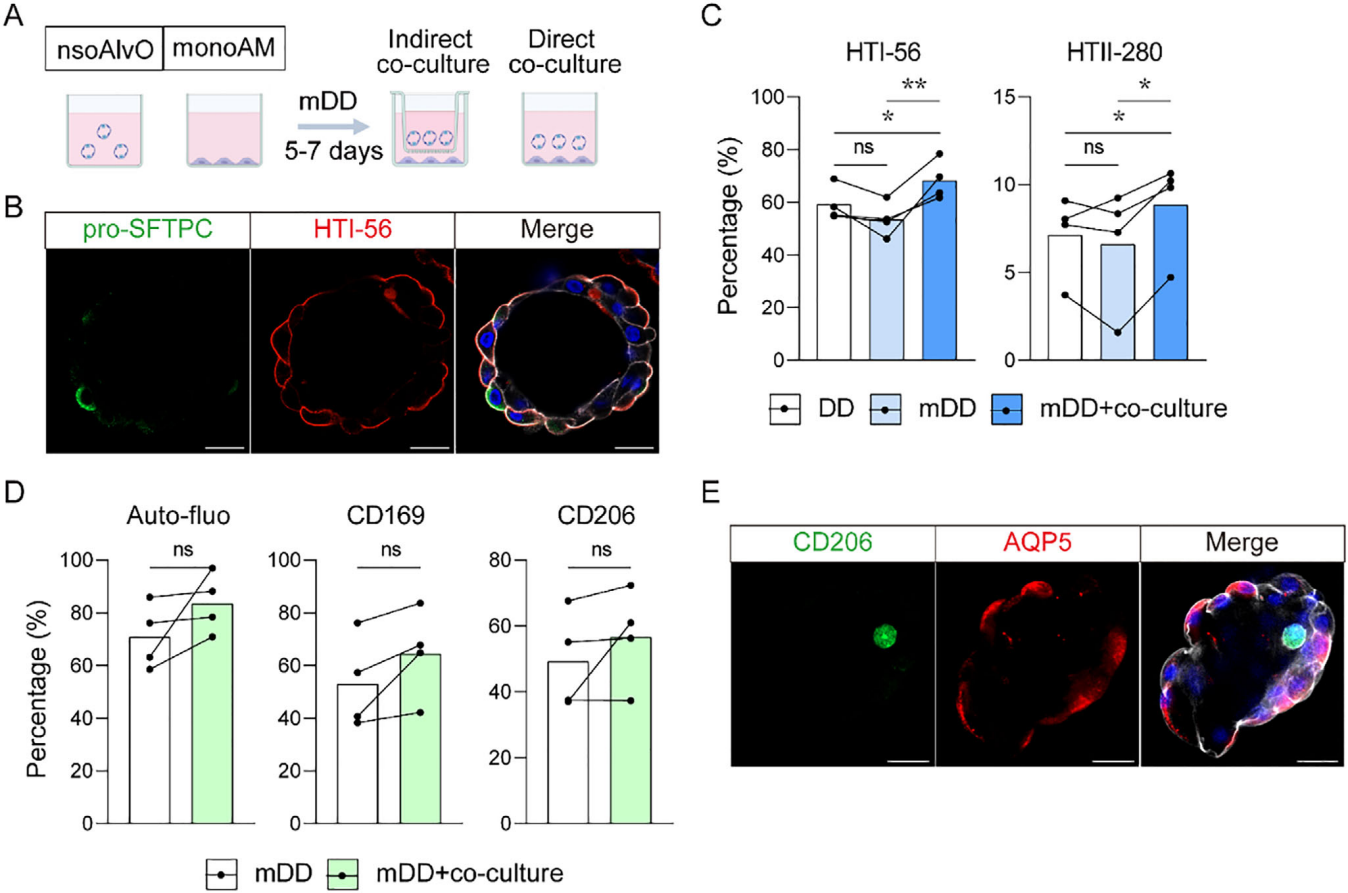
Generation and characterization of the organoid‐macrophage co‐culture and assembloids. (A) Schematic overview of the indirect or direct co‐culture of nsoAlvO and monoAM using mDD medium. Transwell inserts were used for indirect co‐culture. (B) Confocal images of indirectly co‐cultured nsoAlvO labeled with AT1 marker HTI‐56 (red) and AT2 marker pro‐SFTPC (green), counterstained with DAPI (blue) and Phalloidin (white). Scale bar: 20 µm. (C) Flow cytometry analysis of the abundance of HTI‐56+ AT1‐like and HTII‐280+ AT2‐like cells in nsoAlvO. N = 4 independent biological replicates. RM one‐way ANOVA with multiple comparisons. (D) Flow cytometry analysis of the abundance of auto‐fluorescent or CD169/CD206+ AM‐like cells in monoAM. N = 4 independent biological replicates. Ratio paired t‐tests. (E) Confocal images of assembloids labeled with AT1 marker AQP5 (red) and AM marker CD206 (green), counterstained with DAPI (blue) and Phalloidin (white). Scale bar: 20 µm.

To gain an in‐depth and comprehensive understanding, we performed a single‐cell RNA sequencing analysis of organoid‐macrophage assembloids (i.e., direct co‐culture of nsoAlvO and monoAM) in comparison with the undifferentiated nasal cell‐derived organoids (NsO). We identified 7 cell clusters in 2 samples, comprising a total of 41,748 cells, including basal, cycling basal, ciliated, secretory, AT2‐like, AT1‐like, and AM‐like cells (Figure 4A and Figure S3A). Classical cell type‐specific markers, such as KRT5/TP63 for basal cells, MKI67/PCNA for cycling basal cells, FOXJ1/SNTN for ciliated cells, SCGB1A1 for secretory cells, SLPI/PLAU for AT2‐like, AQP5/SCNN1A for AT1‐like, and LYZ/PPARG for AM‐like cells, were abundantly and specifically expressed (Figure 4B and Figure S3B,C). AT2‐like cells expressed PLAU, which is essential for AT2‐mediated alveologenesis [60]. AM‐like cells expressed both M1 (CD86/HLA‐DRA) and M2 (MRC1/CD163) markers (Figure S3C), consistent with the hybrid phenotype of native alveolar macrophages [55]. Differentially expressed gene (DEG) analysis identified the top differentially expressed genes for each cluster, which validated the cell type annotation (Figure S3D). To further evaluate the physiological relevance of the AT1‐ and AT2‐like cells, we analyzed the expression of an array of canonical and enriched markers in native human alveolar epithelial cells [61]. These marker genes were highly enriched in AT1‐ and AT2‐like cells compared to other airway epithelial cell types (Figure S3E), which demonstrate the resemblance of AT1‐ and AT2‐like cells to the native counterparts.

**FIGURE 4:**
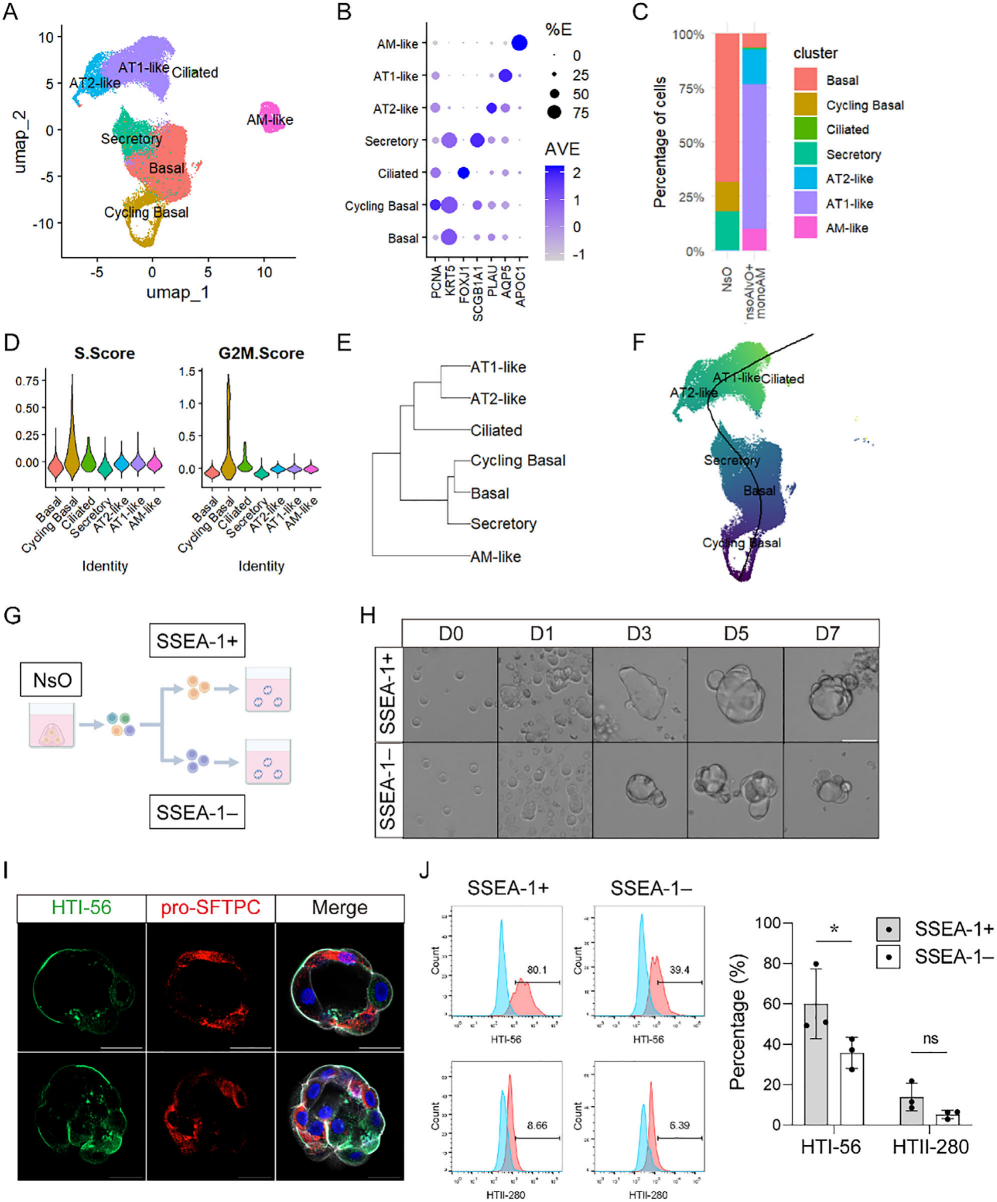
Single‐cell transcriptomic analysis of the nasal cell‐derived organoids and organoid‐macrophage assembloids. (A) UMAP visualization of cells. Each dot represents a cell, and each color represents a cell type. (B) Dot plot shows the percentage expressed (%E) and average expression level (AVE) of cell type‐specific marker genes across clusters. (C) Bar chart shows the percentage of cell types in each sample. (D) Violin plots of cell cycle scores of each cell type. (E) Phylogenetic analysis by building a cluster tree. (F) Phylogenetic analysis by pseudotime trajectory inference. (G) Schematic overview of FACS sorting of SSEA‐1+/– cells from NsO for downstream differentiation. (H) Brightfield images of the isolated SSEA‐1+ and SSEA‐1– cells cultured in DD+ds medium from day 0 to 7 (D0 to D7). Scale bar: 50 µm. (I) Confocal images of organoids derived from SSEA‐1+ cells labeled with AT1 marker HTI‐56 (green) and AT2 marker pro‐SFTPC (red), counterstained with DAPI (blue) and Phalloidin (white). Scale bar: 20 µm. (J) Flow cytometry analysis of the abundance of HTI‐56+ and HTII‐280+ cells in the SSEA‐1+ and SSEA‐1– cell‐derived organoids. Representative histograms are shown on the left. Data represent mean ± SD, N = 3 independent biological replicates. Unpaired t‐tests.

The undifferentiated NsO comprised progenitor cells, including cycling basal, basal, and secretory cells, which revealed the cellular basis sustaining the long‐term expandability of these undifferentiated organoids (Figure 4C). In contrast, organoid‐macrophage assembloids were mainly composed of AT1‐like (66.7%), AT2‐like (15.5%), and AM‐like cells (10.0%) (Figure 4C), consistent with the cellular composition shown above by flow cytometry and confocal imaging (Figures 1 and 3). Cell cycle analysis and scoring revealed the proliferative and progenitor activity of cycling basal cells (Figure 4D). Cluster tree analysis showed a close phylogenetic relationship between progenitor cells (including cycling basal, basal, and secretory cells), as well as alveolar cells (AT2‐ and AT1‐like cells) (Figure 4E).

Pseudotime trajectory analysis indicated the differentiation trajectory and suggested that secretory cells, mainly SCGB1A1+ club cells, in the nasal cell‐derived organoids (NsO) were the direct progenitors of AT2‐like and AT1‐like cells (Figure 4F). SSEA‐1 (CD15) was identified to be a surface marker of progenitor cells co‐expressing both club and AT2 cell markers in neonatal and adult mouse lungs [27]. We verified the co‐expression of SSEA‐1 and club cell marker CC10 in the undifferentiated NsO by immunostaining and flow cytometry analyses (Figure S4A,B); thus, SSEA‐1 was used as a surface marker for cell sorting (Figure 4G). SSEA‐1+ cells were subjected to distal differentiation in the DD+ds medium for 1 week, resulting in the formation of organoids with alveolar‐like morphology; whilst SSEA‐1– cells displayed a notably lower organoid‐forming efficiency (Figure 4H). We observed the presence of HTI‐56+ AT1‐like and pro‐SFTPC+ AT2‐like cells in the SSEA‐1+ cell‐derived organoids (Figure 4I). Flow cytometry analysis revealed that the SSEA‐1+ cell‐derived organoids contained significantly more AT1‐like cells than the SSEA‐1– cell‐derived organoids (Figure 4J). Thus, SSEA‐1+ club cells serve as the primary alveolar progenitor for nsoAlvO.

To better understand the cell type‐specific function and interactions in the assembloids, we performed pairwise DEG, KEGG, and CellChat analyses to identify statistically significant differentially expressed genes, biological pathways, and cell‐cell communication. Oxidative phosphorylation was highly upregulated in AT1‐like cells (Figure 5A), probably due to their high ion transport activity for maintaining liquid homeostasis (Figure 5B). AT2‐like cells displayed highly upregulated pathways related to protein metabolism and homeostasis, such as proteasome, protein processing in the endoplasmic reticulum, endocytosis, and phagosome (Figure 5C), correlating to their function in surfactant metabolism and innate immunity [62, 63]. Tight junction pathway was also highly upregulated in both AT2‐ and AT1‐like cells (Figure 5A,C), consistent with their essential role in the alveolar epithelial barrier. Indeed, tight junction‐related signaling pathways such as JAM and OCLN were highly enriched in both AT2‐ and AT1‐like cells (Figure 5D). Meanwhile, lysosome, phagosome, antigen processing and presentation, and endocytosis pathways were highly upregulated in AM‐like cells (Figure 5E), echoing their role in host defense and maintenance of homeostasis in alveoli. Importantly, epithelial cells, including both AT2‐like and AT1‐like cells, showed extensive intercellular communication with AM‐like cells. AT2‐like cells were one of the top cell‐cell signal senders, while AM‐like cells were the top signal receiver (Figure 5F). Specifically, epithelial cells transmitted signaling cues to the AM‐like cells, including MIF, annexin, complement, and prostaglandin signaling pathways, which are key axes mediating immune cell recruitment, adhesion, survival, and modulation (Figure 5G). AM‐like cells also demonstrated immune cell‐specific activity, such as CD45 and PECAM1, which regulate their adhesion, migration, and inflammatory modulation (Figure 5H). Overall, we generated and comprehensively characterized a physiologically relevant organoid‐macrophage assembloid model incorporating functional alveolar epithelial cells and alveolar macrophages.

**FIGURE 5:**
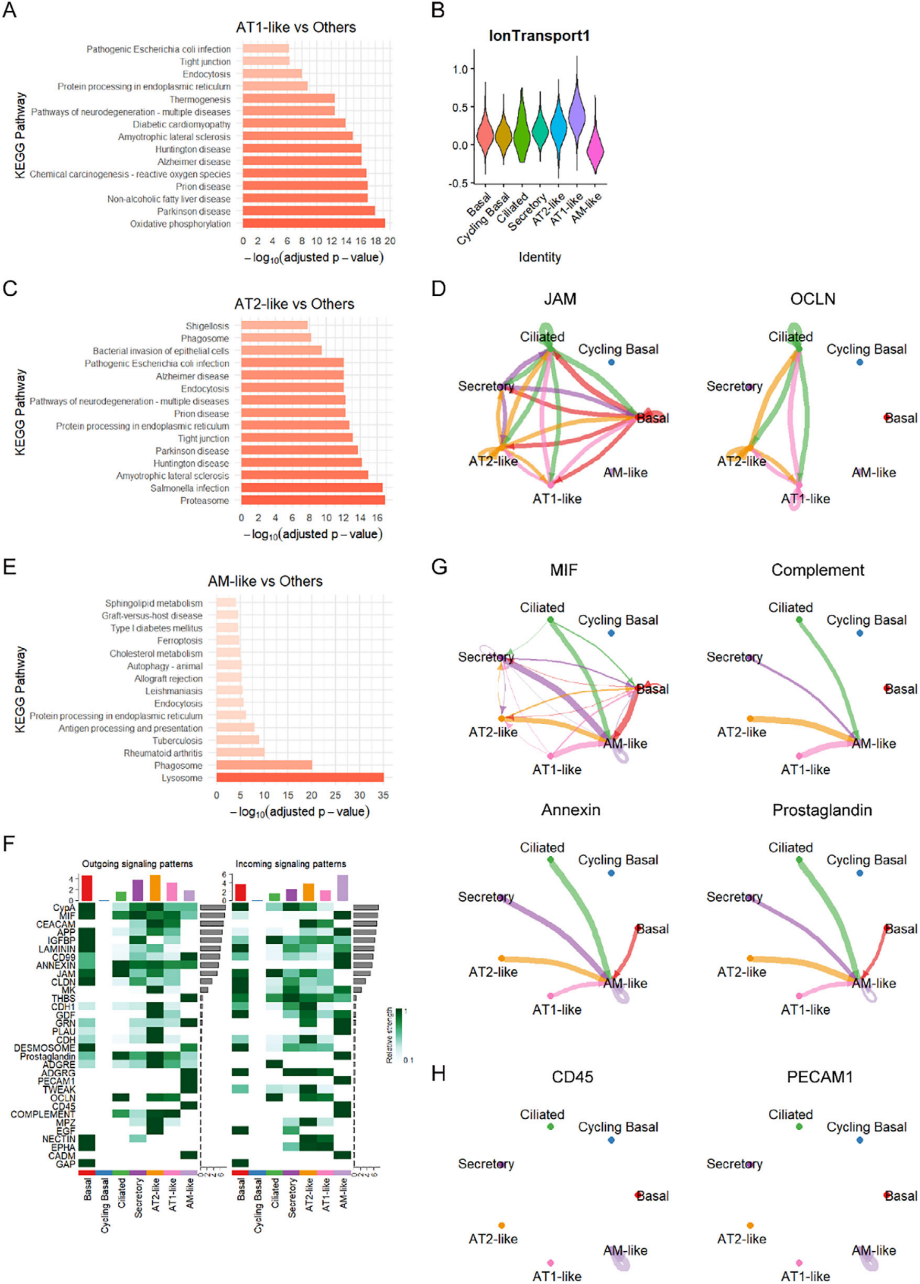
Cell type‐specific biological activities and cell‐cell interactions in the organoid‐macrophage assembloids. (A) Bar chart shows the top upregulated KEGG pathways in AT1‐like cells. (B) Violin plot show ion transport activity score of each cell type. (C) Bar chart shows the top upregulated KEGG pathways in AT2‐like cells. (D) Circle hierarchical plots of the JAM and OCLN signaling pathway network. (E) Bar chart shows the top upregulated KEGG pathways in AM‐like cells. (F) Heatmap shows the top identified cell‐cell communication pathways in the organoid‐macrophage assembloids. (G) Circle hierarchical plot of the MIF, Annexin, Complement, and Prostaglandin signaling pathway network. (H) Circle hierarchical plot of the CD45 and PECAM1 signaling pathway network.

### 2.4 Infection of Influenza A Viruses in Lung Tissue‐Derived and Nasal‐Derived Alveolar Organoids

Influenza A viruses are a large family of negative‐sense RNA viruses that infect human respiratory cells and cause symptoms such as fever, cough, sore throat, and even pneumonia. These viruses exhibit different transmissibility and pathogenicity, primarily due to their differential infectivity and replicative capacity in the upper and lower respiratory tract. For example, highly pathogenic avian influenza H5N1 viruses exhibit higher tropism in the lower respiratory epithelium than human H1N1 viruses [64]. Here, we examined the infection of an avian H5N1 virus and a human H1N1 in AlvO and nsoAlvO. The apical‐out polarity of organoids allows for infection from the apical side, recapitulating real‐life infection in the respiratory epithelium. Both virus strains efficiently infected AlvO, as shown by the immunostaining of viral nucleoprotein (NP) (Figure 6A). There were more abundant NP+ cells with higher fluorescent intensity in H5N1‐infected AlvO than in H1N1‐infected AlvO (Figure 6A). Both HTI‐56+ AT1‐like and non‐AT1 cells appeared to be infected by the viruses (Figure 6A). Quantification of the viral titers in the culture supernatant showed that the H5N1 virus replicated to a significantly higher titer than the H1N1 virus in AlvO (Figure 6B). The differential infectivity of H5N1 and H1N1 viruses was reproduced in nsoAlvO by immunostaining and replication kinetics (Figure 6C,D). These results indicate higher infectivity and replicative fitness of the H5N1 virus than the H1N1 virus in both AlvO and nsoAlvO, which recapitulate the preferential tropism of the H5N1 virus in the human lower respiratory tract.

**FIGURE 6:**
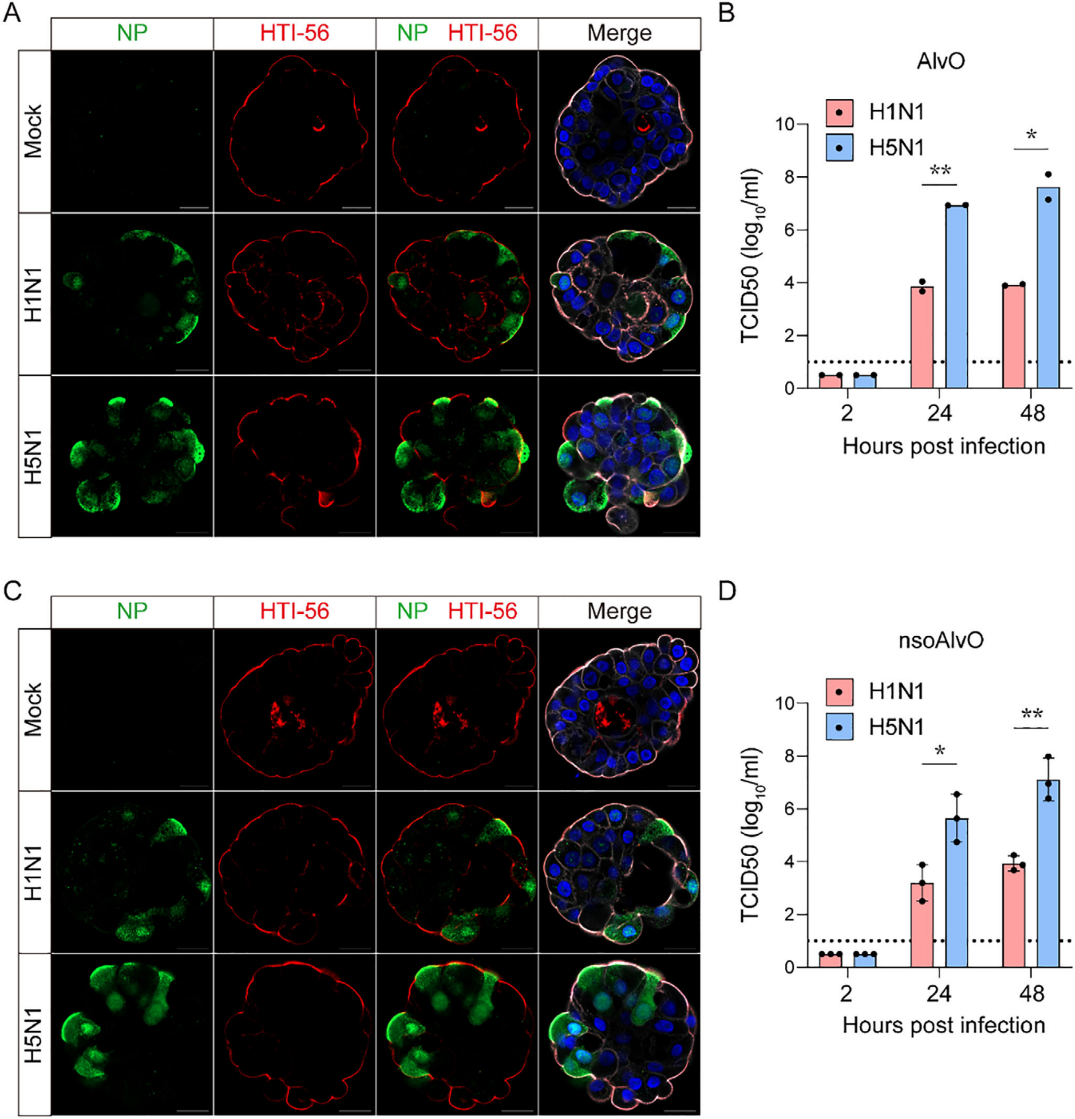
Infection of the tissue‐derived and nasal‐derived alveolar organoids with influenza A viruses. (A,C) At 24 h post‐infection (hpi), influenza A viruses H1N1 or H5N1 (0.1 MOI) infected (A) AlvO or (C) nsoAlvO were harvested and labeled with anti‐NP (green) and anti‐HTI‐56 (red) antibodies to identify virus‐infected cells in the organoids. Scale bar: 20 µm. (B,D) Culture supernatants were harvested from the infected (0.1 MOI) (B) AlvO or (D) nsoAlvO. Virus titers were determined by TCID_50_ assays. Data represent mean ± SD, (B) N = 2 or (D) N = 3 independent biological replicates. Unpaired t‐tests.

We also examined viral infection in organoid‐macrophage indirect co‐culture to decipher the role of alveolar macrophages in pulmonary infection of influenza viruses. The percentage of virus‐infected cells was significantly higher in H5N1‐infected nsoAlvO than in H1N1‐infected nsoAlvO (Figure S5), in line with the imaging results (Figure 6C). Interestingly, the infection rates of both viruses in the nsoAlvO were significantly reduced in the presence of monoAM (Figure S5), suggesting a protective effect of the macrophages against virus dissemination.

## 3 Discussion

Human alveolar sacs, composed of alveolar epithelial cells (AT1 & AT2) and tissue‐resident alveolar macrophages (AM), are the infection sites for highly pathogenic respiratory viruses such as avian H5N1 viruses [65, 66]. However, there has been an unmet demand for a robust, accessible, and physiologically relevant in vitro model of human alveoli. In this study, we established and characterized new human alveolar organoids and organoid‐macrophage assembloids. Briefly, long‐term expandable organoids were derived from readily accessible nasal epithelial cells, in which we induced distal differentiation to generate nasal‐derived alveolar organoids (nsoAlvO) (Figure 1). The nsoAlvO, composed of mostly AT1‐ and AT2‐like cells, adequately recapitulates the morphology, marker expression, and cellular composition of the alveolar epithelium (Figure 1). Importantly, the AT2‐like cells in nsoAlvO exhibit spontaneous uptake and storage of surfactant lipids, one of the characteristics of native AT2 cells in the primary alveoli [30], similar to the AT2 cells within tissue‐derived or PSC‐derived alveolar organoids [7, 10].

Apart from the epithelial cells, the tissue‐residing AM are an indispensable cell population in the alveoli and constitute a significant part of the innate immune system in the lungs. iPSC‐derived isogenic co‐culture models of alveolar organoids and macrophages have been reported [13, 14, 15, 16, 17, 67], yet the identity and role of macrophages have not been examined meticulously. Here, we generated monocyte‐derived alveolar macrophages (monoAM) using monocytes isolated from peripheral blood (Figure 2). The monoAM abundantly expresses human AM‐specific markers and demonstrates significantly enhanced lysosomal, enzymatic, and phagocytic activities compared to the MDM (Figure 2), with a comparable level to the in vivo and ex vivo alveolar macrophages reported previously [68, 69, 70].

The nsoAlvO and monoAM represent accessible and physiologically active models of human alveolar epithelium and alveolar macrophages, respectively. Importantly, the nsoAlvO and monoAM are well adapted to indirect or direct co‐culture; the co‐culture leads to an improved maturation of both components (Figure 3). Single‐cell transcriptomic analysis confirmed the cellular identity of AT1‐like, AT2‐like and AM‐like cells in organoid‐macrophage assembloids (Figure 4). Consistent with previous findings, SSEA‐1+ club cells were found to be the major alveolar progenitor cells for the nasal cell‐derived alveolar organoids (Figure 4 and Figure S4). Moreover, we observed cell type‐specific activities and identified extensive epithelial‐macrophage crosstalk mediated by MIF, annexin, complement, and prostaglandin signaling pathways (Figure 5), some of which have been implicated in epithelial repair or immune regulation. For example, prostaglandin E2 (PGE2) secreted by alveolar epithelial cells upregulates suppressor of cytokine signaling 3 (SOCS3) expression in alveolar macrophages, which in turn dampens pro‐inflammatory JAK‐STAT signaling [71]. Overall, we demonstrate the successful generation of novel organoid‐macrophage assembloids of human alveoli. This is probably the first report that organoids derived from readily accessible nasal epithelial cells have alveolar differentiation potential, which highlights the plasticity of respiratory stem cells.

Through analyzing influenza virus infection, we demonstrate the physiological relevance of these organoids and co‐culture models. The H5N1 virus displays a higher infection and replicative fitness than the H1N1 virus in the AlvO and nsoAlvO (Figure 6), which recapitulates H5N1's tissue tropism in the lower respiratory tract [72, 73, 74]. The presence of monoAM alleviates infection of the H5N1 and H1N1 viruses in the nsoAlvO (Figure S5), consistent with previous findings in murine and porcine models [75, 76]. Here, the protective effect of monoAM is likely mediated by secreted signaling molecules such as IFNs and ISGs, since monoAM and nsoAlvO were physically separated in the transwell. Further investigation is needed to decipher the mechanisms of macrophage‐mediated protection comprehensively. Overall, the assembloids adequately recapitulates the high infectivity and pathogenicity of the H5N1 virus in alveoli and underscores the essential role of alveolar macrophages in controlling influenza infection in the lungs.

In summary, we developed novel human alveolar organoids and organoid‐macrophage assembloids for mimicking functional human alveolar epithelial cells and alveolar macrophages. These organoids and assembloids serve as robust, accessible, and biologically relevant experimental models for diverse applications, including, but not limited to, disease modeling, developing therapeutics, and regenerative medicine. The epithelium‐macrophage assembloids can be further advanced by incorporating additional components to recapitulate higher tissue complexity, such as endothelial and adaptive immune cells. Furthermore, the integration of biofabrication technologies, including organoids‐on‐a‐chip and 3D bioprinting, may enhance uniformity and scalability of the assembloids, thereby facilitating high‐throughput applications.

## 4 Methods

### 4.1 Reagents and Tools Table

**Table:** 

Reagent/Resource	Reference or source	Identifier or catalog number
Experimental models		
MDCK	ATCC	CCL‐34
A/Hong Kong/415742/2009	[77]	N/A
A/Vietnam/1194/04	[78]	N/A
Antibodies		
AQP5	Abcam	ab92320
HTI‐56	Terracebiotech	TB‐29AHT1‐56
SFTPC	Sigma–Aldrich	AB3786
HTII‐280	Terracebiotech	TB‐27AHT2‐280
CD169	Abcam	ab199401
CD206	Abcam	ab270634
Influenza NP	Novus Biologicals	NBP2‐16965
SSEA‐1	BD Biosciences	561716
CC10/SCGB1A1	R&D Systems	MAB4218
Oligonucleotides and other sequence‐based reagents		
qPCR primers	This study	Table S1
GEXSCOPE Single Cell RNA Library Kit Cell	Singleron	4180011
Chemicals, Enzymes, and other reagents		
PulmoOrg Respiratory Organoid Expansion Kit	BiomOrgan	A01‐001
Advanced DMEM/F12	Thermo Fisher	12634010
HEPES	Thermo Fisher	15630‐056
GlutaMax	Thermo Fisher	35050061
Penicillin/Streptomycin	Thermo Fisher	15140‐122
TrypLE Select Enzyme (10X), no phenol red	Thermo Fisher	A1217701
Dexamethasone	Tocris	1126
8‐bromo‐cAMP	Tocris	1140
IBMX	Tocris	2845
B‐27	Thermo Fisher	17504044
Primocin	InvivoGen	ant‐pm‐1
CHIR99021	Tocris	4423
Dextran sulfate	Sigma–Aldrich	42867
Poly‐D‐Lysine	Thermo Fisher	A3890401
RPMI 1640 medium	Thermo Fisher	11875093
MEM medium	Thermo Fisher	11095114
FBS	Thermo Fisher	10082147
MEM‐NEAA	Thermo Fisher	11140050
Sodium pyruvate	Thermo Fisher	11360070
GM‐CSF	Peprotech	300‐03
TGFβ	Peprotech	100‐21
DAPT	Sigma–Aldrich	D9542
Y‐27632	Tocris	1254
RNeasy kit	Qiagen	74106
PrimeScript RT master mix	Takara	RR036B

**Table:** 

Reagent/Resource	Reference or source	Identifier or catalog number
TB Green Premix Ex Taq II	Takara	RR82WR
DAPI	Sigma–Aldrich	D8417
Phalloidin‐647	Sigma–Aldrich	65906
β‐Bodipy FL C12‐HPC	Invitrogen	D3792
LysoTracker Red DND‐99	Invitrogen	L7528
Hoechst 33342	Thermo Scientific	62249
CellMask Deep Red plasma membrane stain	Invitrogen	C10046
Cathepsin B Assay Kit	Abcam	ab270772
Fluorescent latex beads	Sigma–Aldrich	L4655
Software		
GraphPad Prism 8.0/9.0	https://www.graphpad.com	
ImageJ	https://imagej.net/ij/index.html	
FlowJo	https://www.flowjo.com/	
ZEN	https://www.zeiss.com/microscopy/en/products/software/zeiss‐zen.html	
Rstudio	https://posit.co/download/rstudio‐desktop/	
Other		
Eclipse TS100 Inverted Routine Microscope	Nikon	N/A
LightCycler 96 Instrument	Roche	N/A
Confocal 980/900/800 microscopes	Carl Zeiss	N/A
FACSymphony A5 SE cell analyzer	BD Biosciences	N/A
FACSAria Fusion cell sorter	BD Biosciences	N/A
Tecnai G2 20 S‐TWIN scanning transmission electron microscope	FEI	N/A

### 4.2 Methods and Protocols

#### 4.2.1 Establishment, Maintenance, and Differentiation of Organoids

The study has been approved by the Institutional Review Board of the University of Hong Kong/Hospital Authority Hong Kong West Cluster (UW13‐364 and UW21‐695). Nasal cell‐derived organoids and lung tissue‐derived organoids were generated using the BiomOrgan's respiratory organoid expansion kit following the product manual.

To differentiate the lung tissue‐derived organoids into alveolar organoids (AlvO) [7, 8], we cultured the dissociated organoids in a distal differentiation (DD) medium (advanced DMEM/F‐12 supplemented with 1% HEPES, 1% GlutaMAX, 1% Penicillin/Streptomycin, 50 nM dexamethasone, 100 µM 8‐bromo‐cAMP, 100 µM IBMX, 2% B‐27, 100 µg/mL Primocin, 3 µM CHIR99021) for 2 weeks in suspension plates in a standard humidified incubator. To differentiate the nasal cell‐derived organoids into nasal‐derived alveolar organoids (nsoAlvO), we cultured the dissociated organoids in DD medium supplemented with 1 mg/mL of dextran sulfate [29] for 1 week and then DD medium for another week. Residual dextran sulfate may interfere with downstream characterization or assessment with RT‐qPCR assay due to its inhibitory effects on polymerases and reverse transcriptases [79]; thus, it needs to be removed by washing. Parental organoids and differentiated organoids were harvested for characterization. Photomicrographs of organoids were acquired using a routine microscope.

#### 4.2.2 Isolation and Differentiation of Peripheral Blood Mononuclear Cells

Peripheral blood was obtained from healthy blood donors at the Hong Kong Red Cross Blood Transfusion Center according to a protocol approved by the Institutional Review Board of The University of Hong Kong/Hospital Authority Hong Kong West Cluster (UW24‐279). Monocytes were isolated from peripheral blood and differentiated into monocyte‐derived macrophages (MDM) according to a well‐established protocol described previously [40]. Briefly, the monocytes were sequentially cultured in a growth medium (GM) (RPMI supplemented with 10% FBS, 1% Penicillin/Streptomycin, 1X MEM‐NEAA, 1X sodium pyruvate) for 1 day and then cultured in a differentiation medium (DM) (GM supplemented with 10 ng/mL GM‐CSF) for 1 week. To further differentiate the MDM into monocyte‐derived alveolar macrophages (monoAM), the MDM were cultured in a mDD medium (DD medium without CHIR99021, supplemented with 10 ng/mL GM‐CSF, 10 ng/mL TGFβ, 10 µM DAPT, and 5 µM Y‐27632) for 2 weeks.

For indirect co‐culture model, nsoAlvO (differentiated in DD+ds medium for 1 week and then DD medium for 1 week) were cultured in the apical chamber of transwell inserts, while monoAM (differentiated in sequentially in GM, DM, and mDD medium for 1 week) were seeded in the basolateral chamber. For direct co‐culture (i.e., assembloid) model, nsoAlvO were added onto the monoAM in the same compartment. The nsoAlvO and monoAM were co‐cultured in mDD medium for 5–7 days. The cell number ratio of macrophages to organoids was approximately 1:3, considering that approximately 1 alveolar macrophage is detected in 3 alveoli [3].

#### 4.2.3 Virus Infection and Detection

Influenza A viruses A/Hong Kong/415742/2009 (H1N1) [77] and A/Vietnam/1194/04 (H5N1) [78] were propagated in MDCK cells. Cell‐free supernatant was harvested, aliquoted, and stored at ‐80°C. Virus titers were determined by plaque assay in MDCK cells. AlvO or nsoAlvO were incubated with viruses at a multiplicity of infection (MOI) of 0.1 for 2 h at 37°C. After inoculation, the organoids were washed and cultured alone or indirectly co‐cultured with monoAM. At the indicated time points, organoids and culture medium were harvested for virus titration, immunostaining, or flow cytometry analysis.

#### 4.2.4 Quantitative Polymerase Chain Reaction

The organoids and macrophages were harvested and applied to RNA extraction, reverse transcription, and qPCR. mRNA expression levels of cellular genes were measured using specific primers (Table S1). mRNA expression levels of cellular genes were normalized with that of GAPDH.

#### 4.2.5 Immunofluorescence Staining

The organoids and macrophages were harvested and applied to immunofluorescence staining to identify cellular composition. To characterize organoids, antibodies against cell type‐specific markers were used to label AT1 (AQP5, HTI‐56), AT2 (pro‐SFTPC, HTII‐280), and club cells (CC10, SSEA‐1). To characterize macrophages, antibodies against CD169 and CD206 were used to label alveolar macrophages. To identify virus‐infected cells, mock‐ or virus‐infected organoids were stained with anti‐influenza NP antibodies. Briefly, samples were fixed, permeabilized, and blocked, followed by primary and secondary antibody incubation, counterstained with DAPI and Phalloidin‐647, and mounted onto glass slides. Confocal images were acquired using a confocal microscope and processed using ZEN software.

#### 4.2.6 Flow Cytometry and Fluorescence‐Activated Cell Sorting (FACS)

The organoids and macrophages were harvested and applied to flow cytometry to quantify cellular composition. For organoids, antibodies against cell type‐specific markers were used to label AT1 (HTI‐56), AT2 (HTII‐280), and club (CC10, SSEA‐1) cells. For macrophages, antibodies against CD169 and CD206 were used to label alveolar macrophages. To quantify virus‐infected cells, mock‐ or virus‐infected organoids were stained with anti‐influenza NP antibodies. Briefly, samples were dissociated into single cells and fixed, followed by primary and secondary antibody incubation. The labeled cells were processed using a cell analyzer. FlowJo software was used for data processing.

To identify the alveolar progenitor cells in NsO, dissociated live cells were labeled with an anti‐SSEA‐1 antibody. The labeled cells were then sorted to obtain SSEA‐1+ or SSEA‐1– population separately using a cell sorter.

#### 4.2.7 Transmission Electron Microscopy

The organoids were harvested and applied to TEM to identify cell‐type specific ultrastructure, as previously reported [7]. Briefly, differentiated organoids were embedded in resin after sequential fixation in 2.5% glutaraldehyde and 1% osmium. The ultrathin sections were stained with uranyl acetate and examined under a transmission electron microscope.

#### 4.2.8 Surfactant Uptake Assay

To assess the functionality of AT2 cells in the organoids, AlvO or nsoAlvO were sequentially incubated with 1 µM β‐Bodipy FL C12‐HPC and 100 nM LysoTracker Red DND‐99. The organoids were then counterstained with Hoechst 33342 and CellMask Deep Red plasma membrane stain and applied to live cell confocal imaging. Alternatively, the organoids were dissociated and applied to flow cytometry analysis.

#### 4.2.9 Detection of Lysosome and Lysosomal Cathepsin B Activity

To assess the lysosomal activity of the macrophages, MDM or monoAM were incubated with acridine orange or magic red according to the manufacturer's protocol. The macrophages were then counterstained with Hoechst 33342 and applied to live cell confocal imaging.

#### 4.2.10 Phagocytosis Assay

To assess the phagocytic activity of the macrophages, MDM and monoAM were incubated with fluorescent latex beads at a 1:400 dilution for 60 min at 37°C. The macrophages were then fixed for confocal imaging or dissociated and fixed for flow cytometry analysis.

#### 4.2.11 Single‐Cell RNA‐Sequencing

Undifferentiated nasal cell‐derived organoids (NsO) and distal differentiated organoid‐macrophage assembloids (nsoAlvO + monoAM) were harvested for scRNA‐seq analysis following the manufacturer's protocol (Singleron). Briefly, the organoids were dissociated and filtered into a single‐cell suspension. The cells were counted and assessed. Single‐cell suspensions were loaded onto a microwell chip using the Singleron Matrix Single Cell Processing System. Barcoding beads were subsequently collected from the microwell chip, followed by the reverse transcription of the mRNA captured to obtain cDNA. After PCR amplification, the amplified cDNA was then fragmented and ligated with sequencing adapters. The scRNA‐seq libraries were constructed according to the protocol of the GEXSCOPE Single Cell RNA Library Kits (Singleron) and then sequenced on an Illumina Novaseq 6000 with 150 bp paired‐end reads (PE150bp).

Data preprocessing was performed using R Studio following the standard scRNA‐seq analysis pipeline. Briefly, cells that expressed fewer than 1000 genes and more than 4000 genes were removed. Then, cells with more than 20% of mitochondrial genes were excluded. The resulting raw unique molecular identifier (UMI) counts in each cell were normalized, integrated, scaled, and followed by PCA analysis. After neighboring and clustering, UMAP and TSNE projections were generated. The expressions of a range of reported marker genes were analyzed to annotate the different cell types in the organoids. We focused on 8 cell types: basal cells (KRT5, TP63); cycling basal cells (KRT5, TP63, MKI67, PCNA); ciliated cells (FOXJ1, SNTN); club cells (SCGB1A1); AT2‐like (SLPI, PLAU); AT1‐like (SCNN1A, AQP5); and AM‐like (LYZ, APOC1, APOE, PPARG). Phylogenetic analyses were conducted by cell cycle scoring, building cluster tree, and pseudotime trajectory inference. DEG analysis identified top differentially expressed genes, KEGG analysis identified top upregulated pathways, and CellChat analysis identified cell‐cell communication.

#### 4.2.12 Statistical Analysis

Statistical analysis was conducted using GraphPad Prism. Either the Student's t‐test or the ANOVA test was used to determine statistical significance as specified in the figure legends. All the experiments were repeated in organoids or macrophages derived from multiple independent biological donors as specified in the figure legends. ^*^ *p* ≤ 0.05, ^**^ *p* ≤ 0.01, ^***^ *p* ≤ 0.001.

## Author Contributions

M.C.C. and J.Z. designed the experiments. M.C.C., S.Z., C.L., Y.Y., Z.W., J.H., W.X., Y.Z., X.Z., Q.L., J.W., Z.Z., Y.L., and X.M. performed the experiments. M.C.C., S.Z., and C.L. analyzed and interpreted the data. J.Z., K.Y.Y., H.C., H.C., and L.H. provided conceptual advice. J.Z., K.Y.Y., and K.K.T. provided financial/resource support. J.Z. and M.C.C. wrote the manuscript.

## Conflicts of Interest

The authors declare a conflict of interest. J.Z., M.C.C., C.L., K.K.T., and K.Y.Y. are listed as inventors on the patent of nasal cell‐derived human alveolar organoids and organoid‐macrophage assembloids (Application No. 63/880,129). J.Z. is the founder of BiomOrgan Limited. All other authors declare no competing interests.

## Supporting information




**Supporting File**: advs77093‐sup‐0001‐SuppMat.docx.

## Data Availability

The scRNA‐seq dataset was deposited in GSE316878 (token: yxizuaggxfmtzsr). The other data that support the findings of this study are available from the corresponding author upon reasonable request.
