# Supplementary material for: Generation of Nasal Cell‐Derived Human Alveolar Organoids and Organoid‐Macrophage Assembloids for in Vitro Lung Modeling
Source: Adv Sci (Weinh). 2026 Aug 11;13(53):e77093. doi: 10.1002/advs.77093 (PMC13460282; doi:10.1002/advs.77093)
Supplement: Supplementary file 1 [file ADVS-13-e77093-s001.docx]

**Generation of nasal cell-derived human alveolar organoids and organoid-macrophage assembloids for *in vitro* lung modeling**

Man Chun Chiu^1,3^, Shuxin Zhang^1^, Cun Li^1^, Yifei Yu^1,3^, Zhixin Wan^1^, Jingjing Huang^1^, Wei Xue^1^, Ying Zhou^1^, Xiaoxin Zhu^1^, Qiaoshuai Lan^1,3^, Jiali Wu^1^, Zijun Zhao^1^, Yuhong Liu^1,3^, Xinjie Meng^1^, Lin Huang^3,5^, Hin Chu^1,2,3,4^, Honglin Chen^1,2,3,4^, Kelvin Kai-Wang To^1,2,3,4^, Kwok Yung Yuen^1,2,3,4,*^, Jie Zhou^1,2,3,4,5,*^

1. Department of Microbiology, School of Clinical Medicine, Li Ka Shing Faculty of Medicine, The University of Hong Kong, Hong Kong, China
2. State Key Laboratory of Emerging Infectious Diseases, The University of Hong Kong, Hong Kong, China
3. Centre for Virology, Vaccinology and Therapeutics, Hong Kong Science and Technology Park, Hong Kong, China.
4. Pandemic Research Alliance Unit, The University of Hong Kong, Hong Kong, China.
5. BiomOrgan Limited, Hong Kong, China.

^*^ Corresponding authors

**Supporting Information**

**Figure S1. Comparison of nasal-derived and tissue-derived alveolar organoids.**

| **A** | Expression of AT1 and AT2 marker genes in nsoAlvO and AlvO. Data represent mean ± SD, N = 4 (nsoAlvO) or N = 2 (AlvO) independent biological replicates. Unpaired t-tests. |
| --- | --- |
| **B** | Representative histograms of the abundance of HTI-56+ AT1-like and HTII-280/Bodipy-HPC+ AT2-like cells in nsoAlvO, AlvO, NsO and LO. |

**Figure S2. Immune and inflammatory baseline of monocyte-derived alveolar macrophages.**

The baseline expression level of cellular response genes in macrophages. Data represent mean ± SD, N = 3 independent biological replicates. Unpaired t-tests.

**Figure S3. Expression of cell-type specific marker genes in each cell type.**

| **A** | UMAP visualization of cells in each sample. Each dot represents a cell, and each color represents a cell type. |
| --- | --- |
| **B** | Violin plots show the expression of airway and alveolar epithelial cell type-specific marker genes across clusters. |
| **C** | Violin plots show the expression of human alveolar macrophage-specific and M1/M2 marker genes across clusters. |
| **D** | A heatmap shows the top 12 DEGs identified in each cell type. |
| **E** | (Top) Dot plots show the percentage expressed (%E) and average expression level (AVE) of alveolar epithelial cell markers/DEGs in AT1- and AT2-like cells versus other airway epithelial cells. (Bottom) Violin plots show the corresponding total DEG scores. |

**Figure S3.**

**Figure S4. Verification of SSEA-1+ as club cells marker.**

| **A** | Confocal images of dissociated nasal cell-derived organoid (NsO) labeled with club cell marker CC10 (green) and SSEA-1 (red), counterstained with DAPI (blue). IgG and IgM staining images are shown as negative control. Scale bar: 20μm. |
| --- | --- |
| **B** | Flow cytometry analysis of the co-expression of CC10 and SSEA-1 in NsO. |

**Figure S5. Infection of the organoid-macrophage indirect co-culture models.**

Mock or infected (0.1 MOI) nsoAlvO with or without monoAM co-culture were harvested at 24 hpi, dissociated, and labeled with anti-NP antibodies to quantify virus-infected cells in the organoids by flow cytometry. Representative histograms are shown on the left. Data on the right represent mean ± SD, N = 3 independent biological replicates. RM one-way ANOVA with multiple comparisons.

**Table S1.** Primers for qPCR assay.

|  | Forward primer | Reverse primer |
| --- | --- | --- |
| AQP5 | GCTCACTGGGTTTTCTGGGTA | TCCATGGTCTTCTTCCGCTC |
| HOPX | GCCTTTCCGAGGAGGAGAC | TCTGTGACGGATCTGCACTC |
| SFTPC | AGCAAAGAGGTCCTGATGGA | CGATAAGAAGGCGTTTCAGG |
| SFTPD | TGGGCTTCCAGATGTTGCTT | CGACACTTTGGCCATTTGGG |
| APOC1 | AGCCGCATCAAACAGAGTGA | TCCTGGGATGTCACCCTTCA |
| APOE | GGGTCGCTTTTGGGATTACCT | GGGTCAGTTGTTCCTCCAGTT |
| PPARγ | TGTGTTTATTCCCATCTCTCCCAA | AGCATGGAATAGGGGTTTGCT |
| GAPDH | GGAGCGAGATCCCTCCAAAAT | GGCTGTTGTCATACTTCTCATGG |
